# Supplementary material for: The interactome of intact mitochondria by cross-linking mass spectrometry provides evidence for coexisting respiratory supercomplexes
Source: Mol Cell Proteomics. 2017 Dec 8;17(2):216–32. doi: 10.1074/mcp.RA117.000470 (PMC5795388; doi:10.1074/mcp.RA117.000470)
Supplement: Supplemental Data [file supp_RA117.000470_133922_0_supp_23978_fzffwf.zip › spectra_annotation/mito_DR_spectra_annotation/64-1-22-1-7-1.pdf]

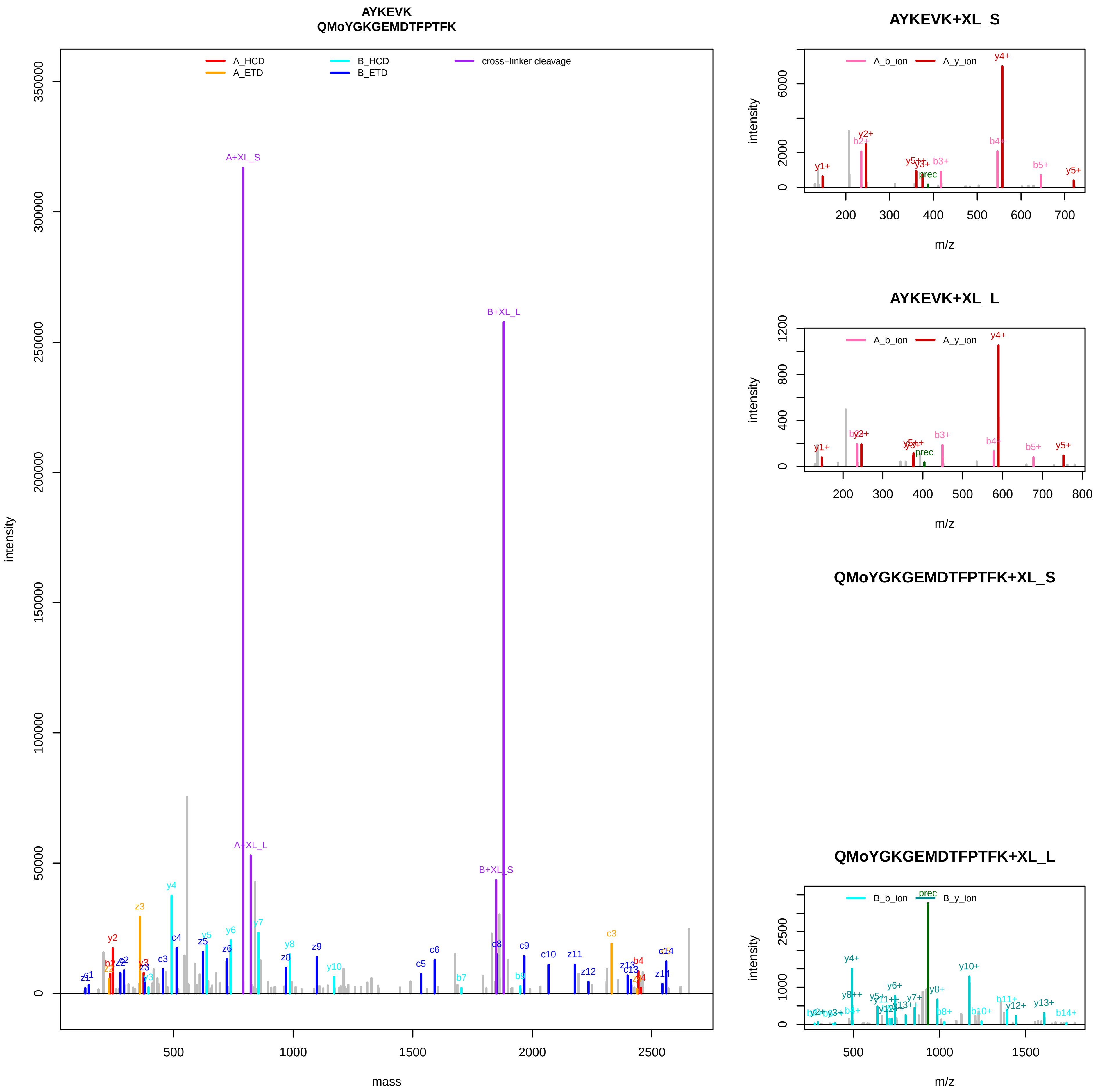
AYKEVK
QMoYGKGEMDTFPTFK
A_HCD
A_ETD
B_HCD
B_ETD
cross−linker cleavage
A+XL_S
B+XL_L
A+XL_L
B+XL_S
intensity
mass
0
50000
100000
150000
200000
250000
300000
350000
500
1000
1500
2000
2500
AYKEVK+XL_S
A_b_ion
A_y_ion
y4+
y2+
b2+
y1+
y5++
y3+
b3+
prec
b4+
b5+
y5+
intensity
m/z
0
2000
6000
200
300
400
500
600
700
AYKEVK+XL_L
A_b_ion
A_y_ion
y4+
y1+
y3+
b3+
prec
b4+
b5+
y5+
intensity
m/z
0
400
800
1200
200
300
400
500
600
700
800
QMoYGKGEMDTFPTFK+XL_S
QMoYGKGEMDTFPTFK+XL_L
B_b_ion
B_y_ion
prec
y4+
y10+
y8++
y6+
y7+
y8+
y11+
y13+
y12+
b14+
b10+
b8+
intensity
m/z
0
1000
2500
500
1000
1500
